# Comparison of sampling methods in assessing the microbiome from patients with ulcerative colitis

**DOI:** 10.1186/s12876-021-01975-3

**Published:** 2021-10-22

**Authors:** Dan Kim, Jun-Young Jung, Hyun-Seok Oh, Sam-Ryong Jee, Sung Jae Park, Sang-Heon Lee, Jun-Sik Yoon, Seung Jung Yu, In-Cheol Yoon, Hong Sub Lee

**Affiliations:** 1 Department of Internal Medicine, Inje University College of Medicine, Busan Paik Hospital, 75 Bokji-ro, Busanjin-gu, Busan, 47392 Korea; 2 Interdisciplinary Program in Bioinformatics, Seoul National University, Seoul, 08826 Korea; 3 ChunLab Inc, Seoul, 06725 Korea; 4 Department of Gastroenterology, Myongji Hospital, Hanyang University College of Medicine, Goyang, Korea

**Keywords:** Colon lavage, Colonoscopy, Inflammatory bowel disease, Microbiome, Ulcerative colitis

## Abstract

**Background:**

Dysbiosis of ulcerative colitis (UC) has been frequently investigated using readily accessible stool samples. However, stool samples might insufficiently represent the mucosa-associated microbiome status. We hypothesized that luminal contents including loosely adherent luminal bacteria after bowel preparation may be suitable for diagnosing the dysbiosis of UC.

**Methods:**

This study included 16 patients with UC (9 men and 7 women, mean age: 52.13 ± 14.09 years) and 15 sex- and age-matched healthy individuals (8 men and 7 women, mean age: 50.93 ± 14.11 years). They donated stool samples before colonoscopy and underwent luminal content aspiration and endoscopic biopsy during the colonoscopy. Then, the composition of each microbiome sample was analyzed by 16S rRNA-based next-generation sequencing.

**Results:**

The microbiome between stool, luminal contents, and biopsy was significantly different in alpha and beta diversities. However, a correlation existed between stool and luminal contents in the Procrustes test (*p* = 0.001) and Mantel test (*p* = 0.0001). The stool microbiome was different between patients with UC and the healthy controls. Conversely, no difference was found in the microbiome of luminal content and biopsy samples between the two subject groups. The microbiome of stool and lavage predicted UC, with AUC values of 0.85 and 0.81, respectively.

**Conclusion:**

The microbiome of stool, luminal contents, and biopsy was significantly different. However, the microbiome of luminal contents during colonoscopy can predict UC, with AUC values of 0.81. Colonoscopic luminal content aspiration analysis could determine microbiome differences between patients with UC and the healthy control, thereby beneficial in screening dysbiosis via endoscopy.

*Trial registration*: This trial was registered at http://cris.nih.go.kr. Registration No.: KCT0003352), Date: 2018–11-13.

## Background

Inflammatory bowel diseases (IBD), which include ulcerative colitis (UC) and Crohn's disease (CD), are chronic intestinal diseases [1]. The patients with IBD have a dysbiosis in the intestinal microbiome [2]. The precise mechanism of the disease has not been elucidated, but microbial dysbiosis is one of its major causes [3]. And as the effect of FMT for IBD treatment has been reported, the need for research on the microbiome composition of IBD is emerging. [4] Currently, stool is the most common evaluation sampling measure for assessing gut microbial communities. Although the stool sampling method is readily accessible, people tend to be reluctant to handle their stool, and the sample can be easily contaminated at home.

The gut microbiome consists of two separate populations, namely, the luminal microbiome and mucosa-associated microbiome. A recent study noted marked heterogeneity of the colonic mucosa, indicating that the microbiome between the stool and mucosal biopsy is different [5]. The mucosa-associated microbiota may have more effect on epithelial and mucosal function than luminal bacteria. Hence, fecalysis may inappropriately represent mucosa-associated bacterial populations [6]. However, the optimal sampling of mucosa-associated microbiome has been seldom investigated.

Mucosal tissue collection through endoscopy is the most ideal method for mucosa-associated microbiome [7], but it is invasive and requires several steps. Therefore, an easy method for evaluating intestinal microorganism imbalance is needed. Recently, colonoscopy has been increasingly used for colon cancer screening. Especially, in patients with UC, colonoscopy was frequently performed for assessing disease activity or treatment response. Aspiration of intestinal fluid through colonoscopy is easy and non-invasive. In general, colonoscopic laxatives exert some influence on the intestinal microbial community [8]. In one study, a colonoscopic laxative significantly reduced the intestinal bacterial count, but in most cases, the count resumed within 14 days [9]. Most of the studies were conducted by collecting stools immediately after colonoscopy rather than examining the fluid aspirated from the colon. According to several studies that applied next-generation sequencing, luminal fluid obtained through colonoscopy is similar to mucosa-associated microbiome [10]. Colonic luminal samples after bowel preparation provide a relatively accurate representation of biopsy microbiome composition and should be considered when biopsy size becomes an issue [11]. Another study using local lavage technique showed similar networks of co-occurring and co-exclusive microbiota, confirming shared features detected in the stool and mucosal lavage compartments [12, 13]. However, analysis of microbiota using local lavage technique in patients with UC has not been reported.

We hypothesized that luminal contents including loosely adherent luminal bacteria after bowel preparation may be suitable for diagnosing the dysbiosis of UC. Therefore, this study assessed the microbiome differences of colonic luminal samples, biopsy, and stool for all participants. For diagnosing the dysbiosis of UC, all of sampling methods were compared between patients with UC and healthy individuals.

## Methods

This research is a prospective, matched, controlled study that was participated by patients scheduled to receive colonoscopy at teaching hospital in South Korea. The institutional review board of hospital approved the study protocols, following the ethical guidelines of the Declaration of Helsinki. This trial was registered at http://cris.nih.go.kr.

This study examined patients with UC and sex- and age-matched healthy individuals. These participants donated stool samples 3 days before colonoscopy and underwent lavage sampling and endoscopic biopsy during the colonoscopy. We then determined whether the three sampling methods had any difference. All colonoscopies were performed by one board-certified, experienced endoscopist (H.S.L.), who has performed > 5000 colonoscopies.

The same bowel cleansing product, that is, 2L of polyethylene glycol (PEG) plus ascorbate solution (Coolprep®; Taejoon Pharmaceuticals, Seoul, South Korea), was given to all participants. All of them were scheduled for an evening session (after 14:00). Three days before colonoscopy, they were instructed to refrain from eating foods with high fiber, seeds, sea algae, and mixed grains. One day before the examination, they were asked to eat light dinner and then fast, except drinking water. On the day of the procedure, they were requested to drink the bowel cleansing product 4–8 h before the examination.

### Subjects

Patients with UC who visited the gastroenterology department of teaching hospital were screened for this study. They were all diagnosed with UC according to the consensus guidelines of the European Crohn's and Colitis Organization [14]. Clinical data such as medical records, blood tests, colonoscopy with biopsy, and radiologic studies were comprehensively reviewed. Furthermore, the clinical activities were evaluated using the Mayo score [15] for UC. Meanwhile, we excluded patients (1) with severe comorbidities, such as cardiovascular diseases, chronic renal diseases, or chronic liver diseases; (2) with mental illnesses; (3)with pregnancy; and (4) with a recent history of gastrointestinal surgery (< 3 months).

The healthy-control group consisted of subjects without gastrointestinal diseases or recurrent abdominal symptoms. They were age- and sex- matched with the patient group. To exclude the presence of any recurrent abdominal symptoms and organic diseases, we let them complete the symptom questionnaire, blood tests, and colonoscopy. In addition, all participants were taught on bowel preparation by well-trained nurses from the gastroenterology endoscopy center. Of note, all of them gave written informed consents prior to participation.

## Experiments

### Sample collection

Using a stool sample collector kit (Chun Lab, Inc., Seoul, South Korea), the subjects provided stool samples 3 days before colonoscopy. At the same time, two pellets (approximately 400 mg each) of stool samples were collected from each subject. Each pellet was placed in two sterile plain tubes without any chemical additives. The stool samples were then stored at − 80 °C before use.

**Fig. 1:**
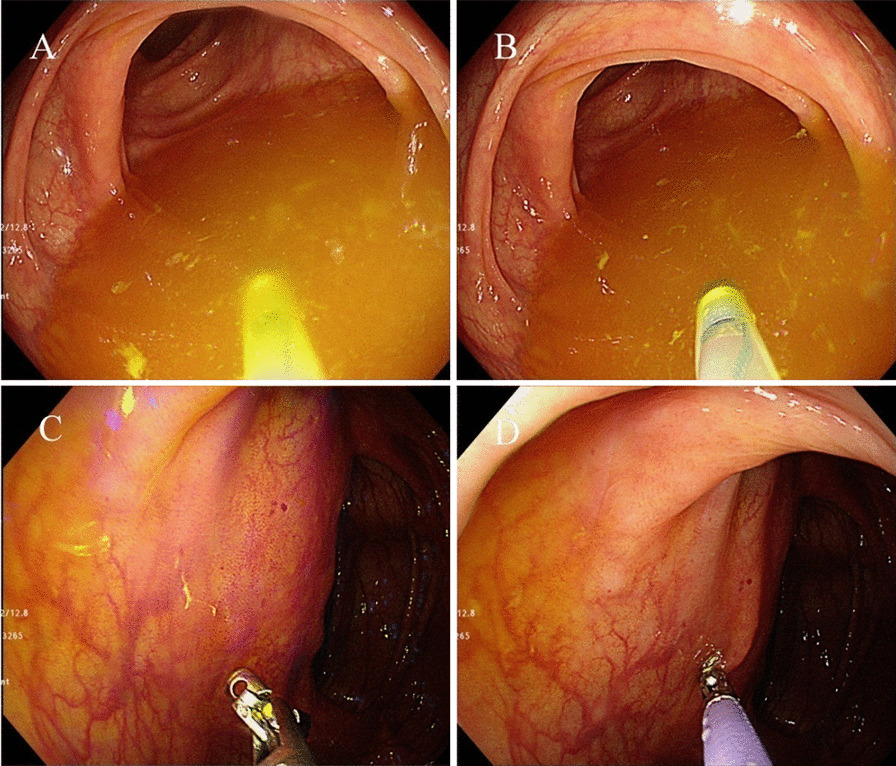
Method to sample the lavage fluid and biopsy. **a**, **b** About 5 ml of colonic lavage fluid in sigmoid colon was collected by using a Clear-Hemostat catheter. **c**, **d** After colonic lavage sampling, the single mucosal biopsy was done at S colon with endoscopic forceps

All endoscopic procedures were performed using a white-light endoscope with colonoscopy (CF-H290; Olympus, Tokyo, Japan). During the colonoscopy, approximately 5 mL of lavage fluid was collected in the sigmoid colon by using a Clear-Hemostat catheter (FM-EH0001, Finemedix Co., Daegu, South Korea) (Fig. 1). At the same time, single mucosal biopsy was performed at the sigmoid colon by using endoscopic forceps (FB-24 K-1; Olympus, Tokyo, Japan). The biopsy sample was kept on ice immediately after collection and then transferred to Chun Lab, Inc. (Seoul, South Korea) on the same day for processing.

### Bacterial community analysis

#### Genomic DNA extraction and amplicon sequencing

400 mg of stool, lavage, and biopsy samples were added to 15 mL of DNA extraction lysis buffer (SDS 4%, Tris–HCL 50 mM, EDTA 50 mM, NaCl 5000 mM) followed by vigorous homogenization by vortexing for 1 min. 1.4 mL of homogenized fecal suspensions were transferred to a 2 mL eppendorf tube and followed by bead beating for 50 s and centrifuging at 14,000 g for 10 min. 200 uL of supernatants were transferred to 96 well plates were kept at − 25 °C until performing PCR. DNA amplification targets V3-V4 regions of the bacterial 16S rRNA gene using 341F and 805R primers. PCR products were sequenced using an Illumina Miseq sequencing system (Illumina, USA) at Chunlab Inc (Seoul, South Korea).

#### Sequencing data processing and statistical analysis

EzBioCloud’s Microbiome Taxonomic Profiling (MTP) cloud was used to classify sequencing reads to bacterial taxonomy and calculate community composition as previously described [16]. For all subsequent diversity and statistical analyses, the classified reads were randomly rarefied to 8,690 reads, which was the minimum read count among all samples. Alpha-diversity (number of OTUs, Shannon diversity, and Simpson index) indices were calculated in OTU-level and beta-diversity (Bray–Curtis dissimilarity) were generated in genus-level using EzBioCloud’s MTP. To see the association between microbial composition variation and host metadata, disease state, and sampling methods, permutational multivariate analysis of variance (PERMANOVA) was conducted using adonis function in vegan R package [17]. PERMANOVA is a geometric partitioning of variation across a multivariate data cloud, defined explicitly in the space of a chosen dissimilarity measure, in response to one or more factors in an analysis of variance design [18]. Pairwise comparisons of community structures between different sampling methods were performed with Procrustes analysis using procrustes and protest function in vegan. The LDA Effect Size (LEfSe; Linear Discriminant Analysis Effect Size) algorithm [19] was used to compare the abundance of taxa among the different groups. Kruskal–Wallis test Pairwise comparisons of community structures between different sampling and Wilcoxon rank-sum test with a significance level of alpha = 0.05 was used [20]. To evaluate the diagnostic ability of bacterial composition for predicting ulcerative colitis, we constructed L1 penalized least absolute shrinkage and selection operator (LASSO) logistic regression model [21] implemented in ‘scikit-learn’ package in python [22]. The read counts were centered log-ratio (clr) transformed [23] and the models were built for each sampling methods and their combinations.

## Results

### Participant's characteristics

From September 2018 to January 2019, we prospectively enrolled 16 patients with UC (9 men and 7 women, mean age: 52.13 ± 14.09 years) and 15 sex- and age- matched healthy individuals (8 men and 7 women, mean age: 50.93 ± 14.11 years). However, one healthy man withdrew the consent. Demographic features including age, comorbidities, and body mass index (BMI) were comparable in both subject groups. Current smoking was significantly lower in patients with UC than that in healthy controls (0% vs. 26.7%; *p* = 0.043). Most of the patients with UC wherein remission, except for four patients with mild to moderate disease activity (Table 1).

**Table.1:** Demographic features of the study subjects

Characteristics	Patients (N = 16)	Controls (N = 15)	*P-*value
Age (year)	52.13 ± 14.09	50.93 ± 14.11	0.816
Body weight (kg)	65.50 ± 9.79	65.07 ± 11.73	0.912
Height (centimeter)	166.63 ± 9.15	166.13 ± 8.46	0.878
BMI (kg/m^2^)	23.60 ± 3.10	23.52 ± 3.67	0.952
Hypertension	4	3	0.539
Alcohol	7	8	0.431
Current smoker	0	4	0.043*
Disease duration (year)			
< 5	6		
5–10	5		
> 10	5		
Disease activity			
Remission	12		
Mildtomoderate	4		
Severe	0		
Location			
E1	9		
E2	4		
E3	3		

Values are presented as mean ± standard deviation or numbers

*Statistically significant

UC: ulcerative colitis; BMI: body mass index; E1, E2,E3: disease localization by Montreal classification

### Comparison between sampling methods for all participants

**Fig. 2:**
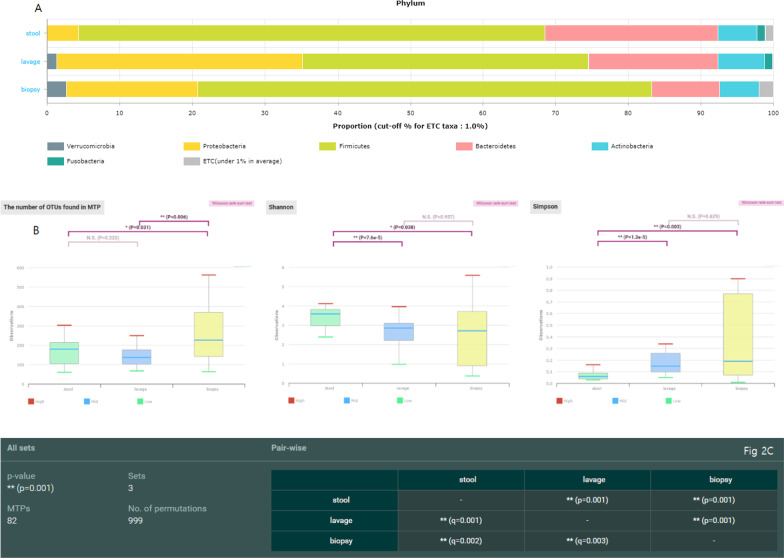
Comparison between sampling methods (**a**) averaged taxonomic compositions at phylum level for stool, lavage, and biopsy sample. **b** Boxplot of Alpha-diversity.in the three study groups stool, lavage, biopsy by means of observed Operational Taxonomic Units (OTUs), Shannon, and Simpson indexes. Plotted in the graphic are the inter-quartile ranges (IQRs) and boxes, medians (lines in the boxes), and lowest and highest values for the first and third quartiles. Each phenotypic category is identified by colors, as indicated on the right side of the figure. Every sample is represented by a colored dot. Solid lines and asterisks indicate that the numbers of OTUs in biopsy sampling was significantly higher than stool sample (*p* = 0.031) and lavage (*p* < 0.006). In the diversity of Shannon and Simpson, stool sample was significantly different with biopsy and lavage sample. **c** Boxplot of Beta diversity (stool, lavage, biopsy). All sampling method in the Beta-diversity was significantly different with each other

Out of 93 biopsy samples, five failed to be analyzed because of having a low read count (< 10,000 reads). Except the five samples, the averaged taxonomic compositions of stool, lavage, and biopsy were analyzed (Fig. 2a). In the lavage and biopsy samples, the number of microorganisms that was in close contact with the intestines, including *Proteobacteria* and *Akkermansia* (a genus in the phylum *Verrucomicrobia*), increased. Figure 2b illustrates the alpha diversity between the sampling methods. The number of operational taxonomic units in biopsy sampling was significantly higher than that in stool sampling (*p* = 0.031) and lavage sampling (*p* < 0.006). On the basis of the diversity of Shannon and Simpson, the stool sample was significantly different from the biopsy and lavage samples. Figure 2c shows the beta diversity between sampling methods. All sampling methods in the beta diversity were significantly different from each other.

**Fig. 3:**
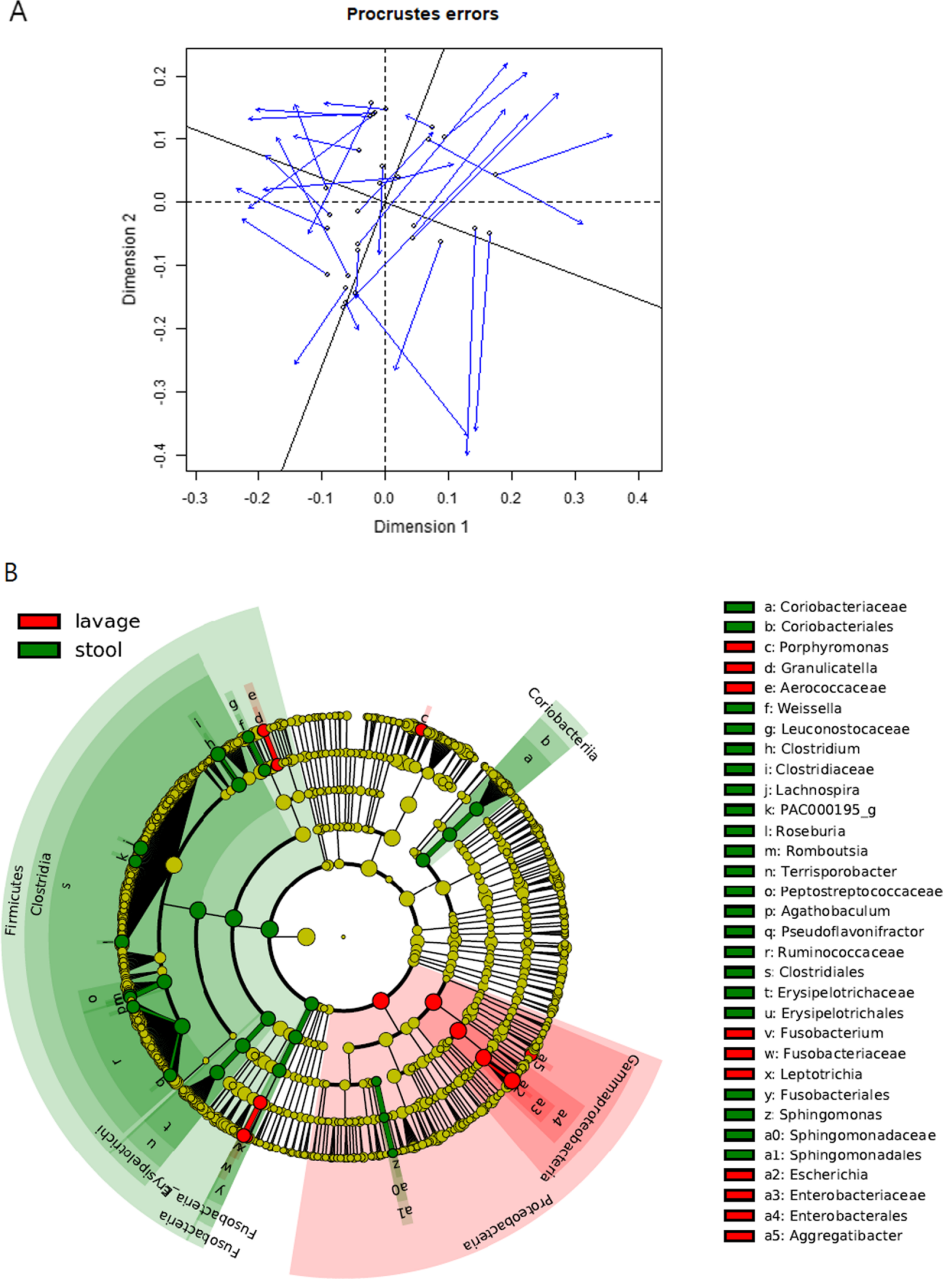
Comparison between colonic lavage and biopsy samples. **a** Procrustes visualizations of non-metric multidimensional scaling plots compared at the phylum and family levels for stool vs. lavage. **b** Cladogram generated by LEfSe indicating differences in taxa between lavage group and stool group. Each successive circle represents a phylogenetic level (phylum, class, order, family, genus). Regions in red indicate taxa enriched in lavage group while regions in green indicate taxa enriched in stool group. Different taxa (at family and order level) are listed on the right side of the cladogram

Table 2 presents the permutational multivariate analysis of variance (PERMANOVA) results between the sampling methods. All sampling methods in PERMANOVA were significantly different from each other. A significant association was also found in the Procrustes test (0.4901, *p* = 0.001) and Mantel test (*p* = 0.0001), indicating a correlation between the stool and wash solution (Fig. 3a). However, the biopsy sample revealed no significant correlation between stool samples (correlation value = 0.2729, *p* = 0.625) and lavage samples (correlation value = 0.2729, *p* = 0.1417) in the Procrustes test (Fig. 3a). The components of the lavage samples showed more proportions of microbiome small intestine (*Proteobacteria* and *Enterobacteriaceae*), oral and the upper GI tract (*Granulicatella*, *Leptotrichia*, *Porphyromonasgingivalis*, *Fusobacterium nucleatum* group, *Parvimonasmicra*, etc.), and respiratory tract (*Corynebacterium durum*) microbiome..compared to those of the stool samples (Fig. 3b).

**Table.2:** PERMANOVA of stool, lavage, and biopsy

		df	pseudo-F	*r*^*2*^	*P-*value
PERMANOVA		2	3.589944	0.094249	0.001
Colon tissue	Lavage	1	2.444345	0.056264	0.013
	Stool	1	2.435198	0.056065	0.001
Lavage	Stool	1	5.339986	0.087056	0.001

df: degrees of freedom; PERMANOVA: permutational multivariate analysis of variance

### Comparison between patients with UC and the healthy control

**Fig. 4:**
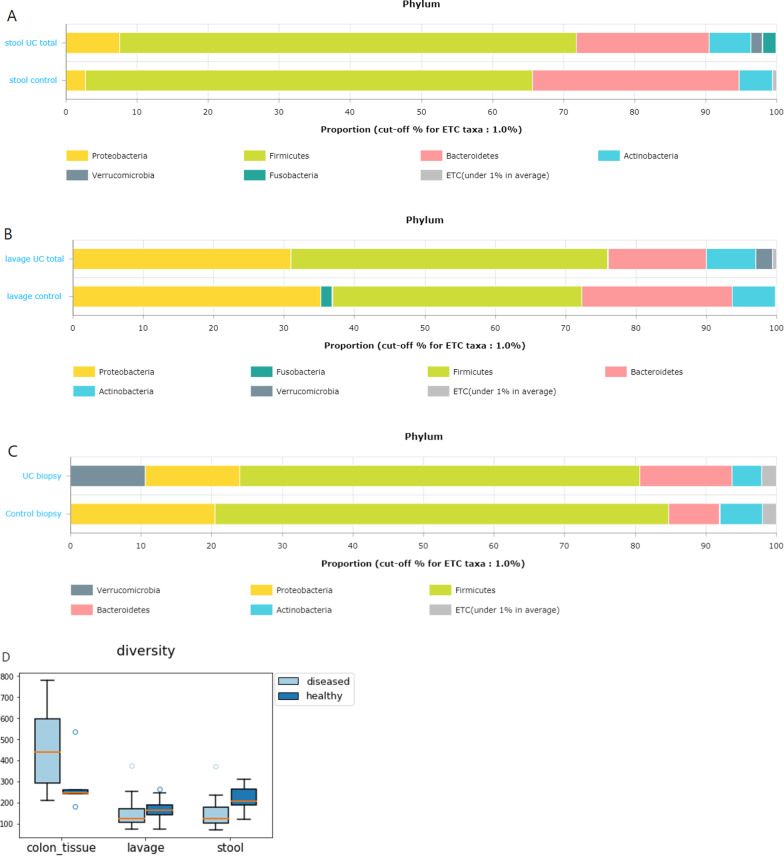
The comparison between patients with UC and control. **a** Averaged taxonomic compositions at phylum level for stool in patients with UC and control. **b** Averaged taxonomic compositions at phylum level for colonic lavage in patients with UC and control. **c** Averaged taxonomic compositions at phylum level for biopsy in patients with UC and control. **d** Significant differences in diversity (number of phylotypes) between UC and healthy controls was found in stool sample

At the phylum level, the relative abundance of *Verrucomicrobia* was higher in the stool, mucosal, and lavage samples of patients with UC than that of the healthy-control (Fig. 4a–c). A significant difference in diversity (number of phylotypes) between the two subject groups was found in the stool sample (Fig. 4d). Conversely, no significant difference was observed in the examination of microorganism diversity in the wash solution and biopsy sample between the two groups. However, the taxonomic distribution of lavage suggests that the differences in diversity may be seen as the number of sample increases.

PERMANOVA confirmed the association between specific factors and the overall microbiome community variation. In the case of stool, UC existence and higher disease activity were distinguished. Lavage was significantly related to current smoking, intestinal surgery, and intestinal symptoms. Furthermore, colon tissue was significantly related to BMI, surgery, order number, and sampling date (Table 3).

**Table.3:** Metadata association with microbiota composition variation (Bray–Curtis distance, Genus level) by permutational multivariate analysis of variance

	df	Stool			Lavage			Colontissue		
		pseudo-F	*r*^*2*^	*P-*value	pseudo-F	*r*^*2*^	*P-*value	pseudo-F	*r*^*2*^	*P-*value
Birthmonth	1	0.496807	0.017434	0.963	0.30401	0.010741	0.988	0.893998	0.064344	0.587
Bristol stool	1	1.615966	0.054564	0.09	1.126012	0.03866	0.338	0.591071	0.04349	0.923
Complication	1	0.978535	0.070003	0.404	1.660764	0.11328	0.132	0.835214	0.094532	0.66
Drinking	1	0.904672	0.031298	0.532	0.629747	0.021996	0.741	0.810813	0.058709	0.709
Extraintestinal	1	1.317045	0.091991	0.133	2.485206	0.160489	0.02	0.928086	0.103951	0.494
Fermentedfood	1	0.77691	0.026998	0.666	1.408026	0.047879	0.159	1.137492	0.080459	0.284
Surgery history	1	0.850101	0.029466	0.567	1.764313	0.059276	0.079	1.232748	0.086613	0.191
Age	1	1.33408	0.045479	0.169	1.187432	0.040683	0.266	0.671462	0.049114	0.876
Birthyear	1	1.341552	0.045722	0.17	1.239168	0.04238	0.226	0.662354	0.04848	0.899
BMI	1	1.382341	0.047047	0.17	0.595062	0.02081	0.798	1.964587	0.131282	0.015
Hostcategory	1	2.348238	0.077376	0.013	1.072956	0.036906	0.31	1.07396	0.076308	0.336
Height	1	1.610117	0.054377	0.085	0.387902	0.013664	0.954	0.483476	0.035857	0.981
Sex	1	0.943593	0.032601	0.467	0.733253	0.025519	0.651	0.707142	0.051589	0.852
Weight	1	1.24015	0.042413	0.237	0.591836	0.020699	0.796	1.821861	0.122917	0.025
Hypertension	1	0.428682	0.015079	0.986	0.724504	0.025222	0.662	0.715157	0.052144	0.839
LesionE1, E23	1	0.833943	0.060282	0.646	0.556671	0.041062	0.865	0.772521	0.088061	0.731
LesionE1, E2, E3	2	1.534195	0.203631	0.053	0.702836	0.104857	0.775	1.097036	0.23864	0.359
Medication	1	0.662934	0.023129	0.789	0.6435	0.022466	0.727	0.729636	0.053143	0.818
Ordernumber	8	1.273526	0.326669	0.099	1.151589	0.304928	0.215	1.450733	0.446279	0.02
Pastdisease	9	0.751518	0.252718	0.882	0.887925	0.285493	0.683	0.818017	0.44995	0.899
Period	1	0.954267	0.068385	0.507	1.564211	0.107401	0.122	0.927049	0.103847	0.529
Probiotics	1	0.632417	0.022087	0.839	0.587319	0.020545	0.815	1.1144	0.078955	0.289
Probiotics	1	0.763851	0.026556	0.692	0.634697	0.022165	0.729	1.329072	0.092754	0.139
Samplingdate	8	1.273526	0.326669	0.11	1.151589	0.304928	0.228	1.450733	0.446279	0.032
Smoking	1	0.895662	0.030996	0.489	2.138451	0.070954	0.041	0.859489	0.062014	0.663
Stoolcolor	3	1.27103	0.1279	0.178	0.880472	0.092224	0.592	1.086841	0.22864	0.331
Stooltype	5	1.000135	0.172433	0.46	0.722303	0.130797	0.91	1.107801	0.380976	0.275
Sugarydrink	1	1.472612	0.049965	0.117	0.863336	0.029911	0.506	1.126826	0.079765	0.286
Sugarysnack	1	0.816938	0.028349	0.628	1.283086	0.043817	0.248	0.799636	0.057946	0.727
Surgery	5	1.509554	0.239249	0.053	1.723835	0.264236	0.004	1.419619	0.279107	0.046
Active UC	1	2.21661	0.073357	0.024	1.015076	0.034984	0.346	1.061837	0.075512	0.358
UC activity	1	1.965438	0.131332	0.046	0.693475	0.050643	0.692	0.710533	0.081572	0.811

BMI: body mass index; df; degrees of freedom; E1, E2,E3: disease localization by Montreal classification; UC: ulcerative colitis

**Fig. 5:**
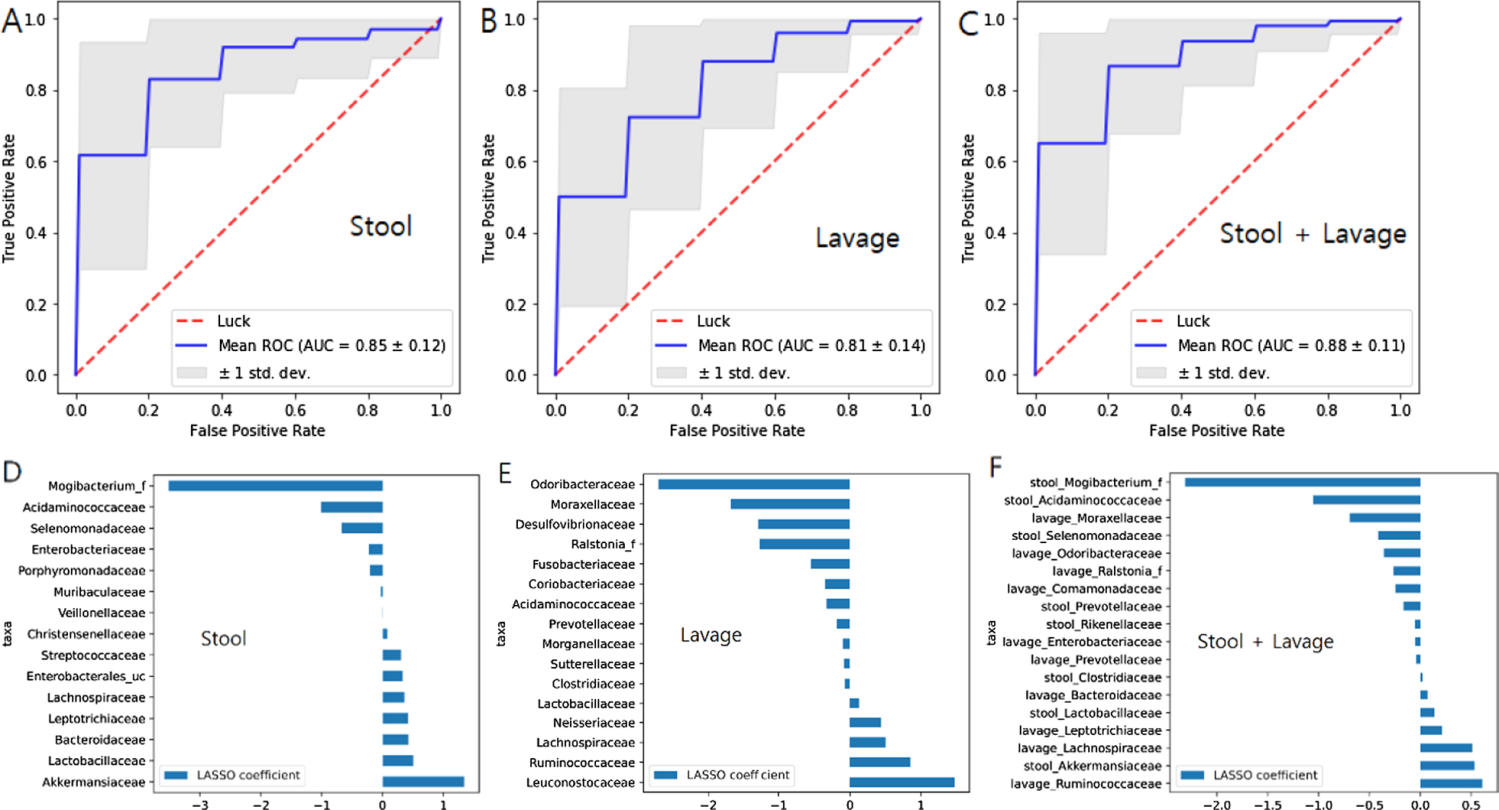
Least absolute shrinkage and selection operator (LASSO) for prediction of ulcerative colitis (UC) means Receiver Operating Characteristic (ROC) curve for UC diagnosis (**a**) Stool predicted UC in accuracy of AUC (The Area Under the ROC curve) 0.85. **b** Colonic lavage predicted UC in accuracy of AUC 0.81. **c** The accuracy of AUC 0.88 increases when using data from stool and lavage. LASSO coefficient was calculated as a method of feature analysis of the LASSO model to find important individual factors. **d** Coefficient plot of LASSO model in stool, e Coefficient plot in lavage, **f** Coefficient plot in stool and lavage

Modeling confirmed that microorganisms in each microbiome can distinguish diseases. Stool and lavage predicted UC, obtaining the AUC values of 0.85 and 0.81, respectively (Fig. 5a, b). The accuracy (AUC = 0.88) increases when using the data from stool and lavage samples (Fig. 5c) LASSO coefficient was calculated as a method of feature analysis of the LASSO model to find important individual factors. (Fig. 5d–f).

**Fig. 6:**
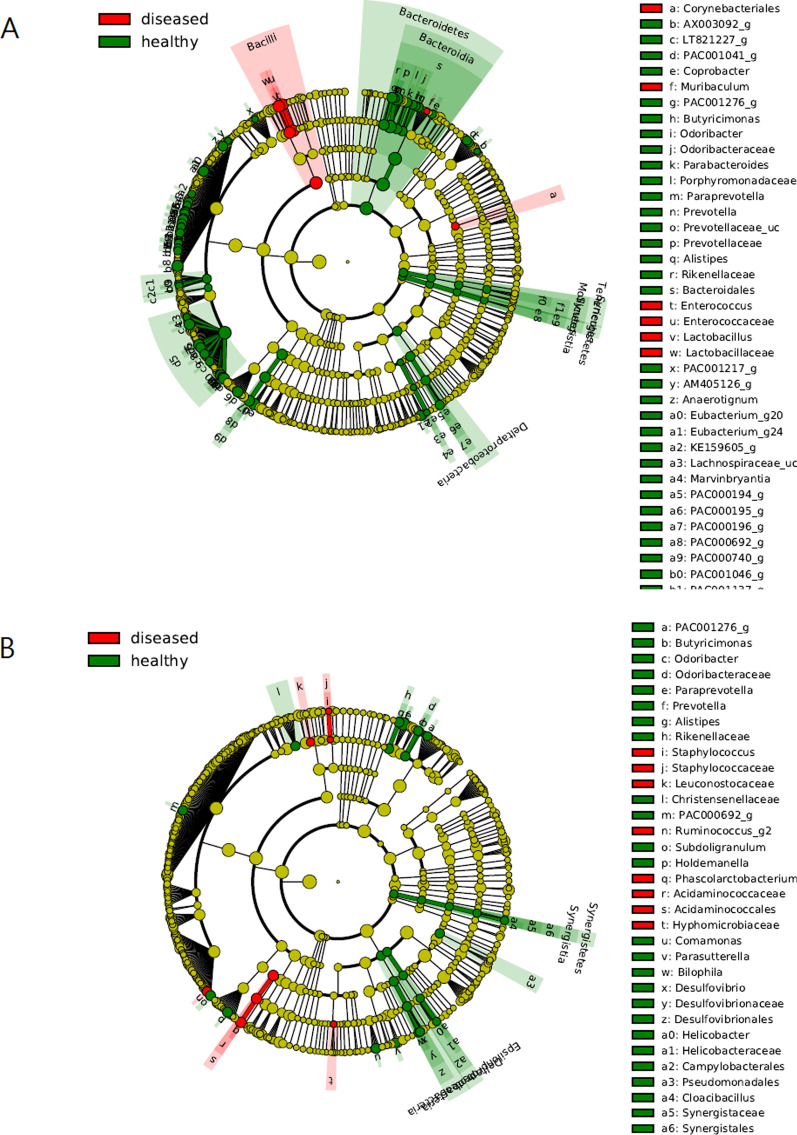
Cladogram generated by LEfSe indicating differences in taxa between ulcerative colitis group and control group. **a** In the case of stool, facultative anaerobes such as *Enterococcus* and *Lactobacillus* are UC-related biomarkers, and several species of *Bacteroidetes* (*Prevotella*, *Bacteoides*, etc.) and *Clostridiales* are found as markers of healthy control. **b** In case of lavage, *Staphylococcus*, *Leuconostocaceae* are the disease markers, and *Prevotella*, *Alistipes*, *Desulfovibrio* are the healthy control markers

In the case of stool, we found that facultative anaerobes such as *Enterococcus* and *Lactobacillus* are UC-related biomarkers, and several genera of *Bacteroidetes (Prevotella*, *Bacteroides*, etc.) and Clostridiales are the healthy-control markers (Fig. 6a). In the case of lavage, *Staphylococcus* and Leuconostocaceae are the disease markers, whereas *Prevotella*, *Alistipes*, and *Desulfovibrio* are the healthy-control markers (Fig. 6b). Therefore, lavage has fewer markers than stool, and microorganisms different from those in the stool are disease and healthy-control markers.

## Discussion

Despite the reduction of beneficial microbes, expansion of pathobionts, and the reduced microbial diversity, which all indicate IBD, the dysbiosis remains inconsistent, and the composition of microbiome varies [24]. The reason for variation may be because of heterogeneity in the sampling method and the disease status of the subject.

In this study, although lavage fluid and stool showed some correlation, all three samples (i.e., lavage, stool, and biopsy samples) were significantly different from each other. The results are consistent with those in previous studies. The microbial communities of multiple biopsy sites within the mouth, stomach, duodenum, and colon were largely distinct from those in stool [5, 25, 26]. In addition, the microbiome variation by colonic biopsy between different colonic regions showed the significant effect by sampling site on the abundance of bacterial taxa [27]. In our study, the biopsy and lavage samples revealed that *Firmicutes* and *Bacteroidetes* were less abundant, with the concomitant increase of *Proteobacteria* and *fusobacteria.* A study participated by Korean patients with CD showed a similar distribution to our study [28]. Thus, biopsy reflects mucosa-associated microbiome, whereas the stool reflects the luminal microbiota in the distal large intestine.

Luminal contents after bowel preparation provide the closest microbiome of the biopsy sample [10]. Colonic lavage samples also provide a relatively accurate representation of biopsy microbiome composition [11]. Bowel preparation appears sufficient to cleanse the lumen to ensure that the luminal contents sampled at colonoscopy—being derived from mucus-adherent microbes rather than the bulk stool stream—contain a microbial community more similar to that of the biopsy samples. The stool material obtained during colonoscopy could be more representative of the mucosal layer. Hence, a noninvasive lavage analysis would be a good alternative method to biopsy. However, in this study, biopsy and lavage samples had no association. Five biopsy samples were not analyzed in this study because of low read counts. The reason is that biopsy procedure is too invasive to acquire enough amount of tissue for microbiome analysis. Furthermore, bowel cleansing washed the entire intestine; thus, the post-bowel preparation fluid could contain loosely attached mucosa-associated microbiome and the whole part of luminal microbiome from the mouth to the colon. The lavage microbes would originate from the whole intestine, oral cavity, and respiratory tract; therefore, colonic lavage samples may have some variances. Meanwhile, another method using local lavage and brush technique for each intestinal part has been reported but still requires validation [12, 13, 29]. In addition, a recent review of the microbiome sampling method urged the development of an ingestible sampling method that can be used in normal people, does not require bowel preparation, and is non-invasive [30].

Most of the studies analyzing the mucosa-associated microbiome performed biopsy sampling after bowel cleansing. In general, colonoscopy preparations can result in multiple negative effects on the intestinal microbiome. Standard bowel preparation can alter the diversity of mucosa-associated microbiota [31]. At the class level, *Proteobacteria* and *Coriobacteria* increase, whereas *Clostridia* significantly decrease after colonoscopy [32]. In normal individuals, a high-volume PEG bowel cleansing preparation has a long-lasting effect on the gut microbiota composition and homeostasis. The short-term effects of bowel preparation may be because most of the bacteria are being washed out in a nondiscriminatory manner, reducing the low-abundance taxonomic groups to levels below detection; on average, bowel preparation with PEG can reduce bacterial load [8]. In fresh stool samples and distal colonic mucosal biopsies collected from 24 healthy subjects before and during a flexible sigmoidoscopy of an unprepared bowel, stool samples obtained a significantly higher diversity than the mucosal samples [33]. More endoscopic studies of an unprepared bowel would be necessary to validate the biopsy method.

The stool samples showed significant differences in diversity between UC and healthy controls. Conversely, the microorganism diversity examination in the colonic lavage and biopsy samples had no significant difference between these two groups. However, the sample size in this study is small to acquire accurate verification. Therefore, we cannot conclude that lavage does not differ between the two groups, considering that the association between intestinal microorganisms and metadata does not consistently appear as a change in the overall population. The change of one or some combinations of specific microorganisms may be a feature that distinguishes UC, and in this case, it can be confirmed by modeling such as machine learning. In this study, stool and lavage predicted UC, obtaining the AUC values of 0.85 and 0.81, respectively, for accuracy. Considering that the accuracy of the model is relatively high (AUC > 0.8), lavage also has a diagnostic potential to verify UC. In another study using biopsy and stool, terminal ileum biopsies performed best (AUC = 0.85), closely matched by the rectum biopsies (AUC = 0.78). The classifier based on the stool samples collected during the diagnosis performed less satisfactorily (AUC = 0.66), with low consistency [7]. Thus, microbiome analysis using colonic lavage can distinguish diseases.

Recently, there was a study comparing the microbiome composition of stool and intestinal lavage fluid of colorectal cancer patients. The study concluded that the microbiome composition of intestinal lavage fluid may be related to the mucosa-associated microbiota associated with carcinogenesis of colorectal cancer [34]. If these similar studies are conducted, it will be possible to prove the assumption that the microbiome obtained from lavage fluid represents a mucosa-associated microbiota, which is associated with pathogenesis.

Facultative anaerobes such as *Enterococcus* and *Lactobacillus* are UC-related biomarkers in the case of stool. This result correlates with the shift of bacterial communities from obligate to facultative anaerobes, strongly suggesting a disruption in anaerobiosis and pointing to a potential role for oxygen in intestinal dysbiosis [35]. Decreased influence of obligate anaerobes and increased influence of facultative anaerobes and some aerobic bacterial communities generally happen in UC [36]. In addition, *Bifidobacterium* and the *Lactobacillus* group were increased in patients with active IBD [37]. And at the species level, Microbial feature of IBD can be considered a decrease in *Faecalibacterium prausnitzii*, increase of *Proteonbacteria* as well as the described increase of *Candida albicans*, *Basidiomycota*/*Ascomycota* ratio over *Saccharomyces cerevisiae* and of the *Caudovirales* over *Microviridae *[38]. Generally, in subjects with infectious diarrhea, *Proteobacteria* and *Enterobacteriaceae* increase and *lactobacilli* decrease [39]. In the case of lavage, *Staphylococcus* and *Leuconostocaceae* are the disease markers. *Staphylococcus* could be one of the candidates involved in IBD pathogenesis. *Staphylococcus aureus* can be detected in a UC-affected colon [40]. The species of the Enterobacteriaceae family only increased in patients with CD; examples were *Escherichia/Shigella* species, which commonly invade the gut mucosal epithelium, causing bloody diarrhea and colonic ulceration [41]. The stool bacteria from patients with UC could cause stronger inflammatory responses than those from healthy controls [42].

A typical gut microbiota in patients with IBD is characterized by a decrease in stool bacteria such as *Firmicutes* and *Bacteroidetes* and an increase in *Proteobacteria*. However, this finding was not significant in this study. Enrolled patients had inactive UC. Hence, their gut microbiome tended to be similar to that of healthy individuals, suggesting that the stool microbiota has different roles in UC pathophysiology [43].

Meanwhile, this study has several limitations. First, the lavage procedure was not performed at each local intestinal part. Some lavage fluid in the sigmoid colon may not represent the whole intestine microbiome status. Nonetheless, the colonic lavage sample can show the whole gastrointestinal mucosa-associated microbiome status. Second, our sample size is small; we only enrolled a limited number to extensively analyze each stool, lavage, and biopsy sample. Third, our study did not demonstrate longitudinal microbiome variation between patients with UC and the healthy control. Hence, further studies with more subjects and longitudinal samplings are necessary.

## Conclusion

In conclusion, the sampling methods of analyzing the colon microbiome differ from each other. Colonoscopic lavage analysis can determine microbiome differences between patients with UC and the healthy control, thereby beneficial in screening dysbiosis via endoscopy.

## Data Availability

All data are fully available without restriction from the EzTaxon-e database (http://eztaxone.ezbiocloud.net).

## Abbreviations

UC: Ulcerative colitis
AUC: Area under receiver operating characteristic curve

## Declarations

### Peer-review

Externally peer-reviewed.
